# Neurodevelopmental follow-up care pathways and processes for children with congenital heart disease in Australia

**DOI:** 10.1038/s41390-024-03722-2

**Published:** 2024-11-23

**Authors:** Bridget Abell, David Rodwell, Karen J. Eagleson, Ben Auld, Samudragupta Bora, Nadine A. Kasparian, Robert Justo, William Parsonage, Steven M. McPhail

**Affiliations:** 1 Australian Centre for Health Services Innovation and Centre for Healthcare Transformation, School of Public Health and Social Work, Faculty of Health, Queensland University of Technology, Brisbane, QLD Australia; 2 Centre for Accident Research & Road Safety—Queensland (CARRS-Q), School of Psychology and Counselling, Faculty of Health, Queensland University of Technology, Brisbane, QLD Australia; 3 Queensland Paediatric Cardiac Service, Queensland Children’s Hospital, Brisbane, QLD Australia; 4 Faculty of Medicine, The University of Queensland, Brisbane, QLD Australia; 5 Health Services Research Center, University Hospitals Research & Education Institute; Department of Pediatrics, University Hospitals Rainbow Babies & Children’s Hospital, Case Western Reserve University School of Medicine, Cleveland, OH USA; 6 Heart and Mind Wellbeing Center, Heart Institute and the Division of Behavioral Medicine and Clinical Psychology, Cincinnati Children’s Hospital Medical Center and the Department of Pediatrics, University of Cincinnati College of Medicine, Cincinnati, OH USA; 7 Department of Cardiology, Royal Brisbane & Women’s Hospital, Herston, QLD Australia

## Abstract

**Background:**

International consensus exists for neurodevelopmental follow-up care of children with congenital heart disease (CHD) to support timely intervention for developmental delays. Yet, documentation of how this care is implemented in Australia is lacking. This study aimed to identify, categorise, and understand care pathways and services supporting neurodevelopmental follow-up of Australian children with CHD.

**Methods:**

A qualitative study, using semi-structured virtual interviews with healthcare professionals across Australia involved in neurodevelopmental care of children with CHD (n = 52) was conducted. Data was analysed using a rapid qualitative approach including structured templates, data reduction, and inductive-deductive analysis of matrices to synthesise data.

**Results:**

Most neurodevelopmental follow-up was delivered as pathways through existing healthcare services rather than centre-based cardiac programmes. Service availability and accessibility varied across the country. Community-based primary care services, paediatric clinics, child development services, neonatal follow-up programmes, and allied health providers were commonly accessed pathway components. However, participants reported a lack of formal structures to coordinate care pathways.

**Conclusions:**

The study identifies how cardiac neurodevelopmental follow-up in Australia can be embedded into existing services and adapted to meet local needs and contexts. Future approaches will benefit from integrating, leveraging, and growing existing services, although adoption of new models may be needed.

**Impact:**

- This study found neurodevelopmental follow-up care for children with CHD in Australia to be delivered as pathways through existing services rather than the centre-based cardiac follow-up programmes common in North America.
- Our study describes alternate options, including providers in community settings, that can be used for follow-up care delivery and how these can adapted to local context.
- Future approaches will benefit from integrating, leveraging, and growing existing services, although adoption of new models may be needed. Greater systematic coordination of care pathways is still required to optimise service delivery, inform planning, and support implementation of national standards of care.

## Introduction

Children with congenital heart disease (CHD) are at higher risk of adverse neurodevelopmental outcomes compared with the general population.^1–3^ Consequently, as increasing numbers of children with CHD survive to adulthood, timely recognition and intervention for those with neurodevelopmental concerns is key to improving long-term outcomes for these children, their families, the health system, and communities.^4^ International consensus exists for longitudinal neurodevelopmental follow-up care for children with CHD, with practice recommendations published by the American Heart Association (AHA),^5^ Cardiac Neurodevelopmental Outcome Collaborative (CNOC),^6^ and Australian Childhood-onset Heart Disease Standards Writing Group.^7^ All recommendations take a risk-stratification approach including routine developmental surveillance and periodic screening for low-risk children, and formalised evaluation of developmental domains for those at highest risk. Children are then connected with the intervention services necessary to address their developmental needs.

Structured cardiac neurodevelopmental follow-up programmes play an important role in translating these recommendations into practice. Longitudinal multidisciplinary follow-up programmes specifically for children with CHD have been established within paediatric surgical centres across the United States^8,9^ and to a lesser extent Canada,^10^ and Europe.^11^ These programmes have been well accepted by families and demonstrate positive outcomes for referral to support services. However, barriers to their uptake and variations in programme implementation have been reported.^12^ Questions also remain about the implementation and feasibility of these mostly centralised and hospital-based programmes across different contexts.^10,13^ For example, research demonstrates an absence of such programmes for children with CHD in South Africa^13^ and the United Kingdom,^14^ which both have a greater reliance on individual paediatric clinicians to perform surveillance and screening. Additionally, sites in Australia and France have adopted alternate models such as decentralising care to multi-disciplinary community based providers,^15^ or using a combination of general paediatrician follow-up and parental screening questionnaires.^16^

It is unlikely that any single care model for cardiac neurodevelopmental follow-up will meet the needs of all families and health services. Some families prefer to return to a familiar hospital for follow-up care, while others may experience emotional and physical stress when making frequent visits to the same hospital.^17,18^ From a health service perspective, different efficiencies and costs are associated with centralising and decentralising care.^19^ Additionally, the role of different implementation contexts must be considered. In regions with a geographically dispersed population, such as Australia, programmes based predominantly at tertiary centres may create inequities in access for those living distant from the follow-up centre.^20–22^ Consequently, a range of approaches may be needed to meet the needs of different families and health systems, and equitably implement recommendations for neurodevelopmental follow-up across different countries and contexts.

The implementation of several individual programmes for cardiac neurodevelopmental follow-up of children in Australia has been reported.^15,23,24^ However, documentation of common approaches to follow-up at a national level is lacking. Given the recent publication of national standards which recommend developmental surveillance and screening for all children with CHD, and formal standardised developmental evaluation for children at high-risk,^7^ it is imperative to more broadly understand how current national practice is supporting optimal follow-up of these children. From a health service perspective, understanding common models of care and follow-up pathways is important for supporting the implementation of these standards, as well as identifying gaps and developing potential strategies to optimise service delivery. Therefore, this study aimed to identify, categorise, and understand existing care pathways and services that support the neurodevelopmental follow-up needs of Australian children with CHD, drawing on the knowledge and experiences of healthcare professionals.

## Methods

### Design

We performed a qualitative, interview-based study to capture information about the models of care supporting neurodevelopmental needs of children with CHD in Australia from a service delivery perspective. This qualitative approach was aligned with both the exploratory nature of the research, as well as our aim to build understanding of current follow-up practices. Additionally, the exploratory descriptive design chosen^25^ allowed for in-depth exploration of the real-world experiences of professionals embedded in healthcare services, including understanding the ‘who’, ‘what’ and ‘where’ of neurodevelopmental follow-up care. The study was informed by a scoping review of international models of care^12^ as a means of understanding the key elements and processes involved in service delivery and to ground the research in existing practice.

Approval was granted by the Children’s Health Queensland Hospital and Health Service Human Research Ethics Committee (LNR/21/QCHQ/73748) prior to study commencement. Reporting of recruitment, data collection and analysis was guided by the COnsolidated criteria for REporting Qualitative Research.^26^

### Sample and recruitment

As this study was concerned with the organisation and delivery of care from a service perspective, healthcare professionals (rather than families or service users) were the target sample. Participants from across Australia with experience in the design, implementation, or delivery of neurodevelopmental care for children with CHD were eligible, including both those with specific cardiac neurodevelopmental experience and others working in cardiology or developmental paediatrics more broadly. We aimed to recruit professionals across a range of geographical locations, clinical settings, disciplines, leadership levels, and years of experience. Consequently, purposeful sampling using a combination of snowball and maximum variation strategies^27^ was employed to identify and recruit participants through existing clinical networks and via study partners based at hospitals across Australia. In this manner the final sample was built by asking for recommendations of new participants with key characteristics from those already interviewed in the study. A combination of thematic saturation (no new information arising from new participants) and data sufficiency across key participant demographics was used to establish the final sample size.^28^

Initially, an introductory email was sent to potential participants by clinical study partners in each Australian state and territory. Those who expressed interest were sent another email by the project co-ordinator to provide participant information and schedule an interview, with a follow-up email to non-responders two-weeks later. All participants were informed that participation was voluntary and would not affect their professional relationships or employment with study partners. Informed verbal consent was obtained prior to starting each interview. The same recruitment and consent procedures were followed for participants identified via snowball sampling.

### Data collection

A semi-structured interview guide (Supplementary Material 1) was developed by BA based on a scoping review of previous literature^12^ and expert opinion of clinical team members. It comprised a set of open-ended questions about current roles and practices in cardiac neurodevelopmental follow-up care and probing questions to elicit detail about specific service elements or map family journeys through existing pathways. Participants’ professions, locations and other demographic characteristics were also collected.

Two experienced PhD-qualified health service researchers (BA, female and DR, male) conducted virtual semi-structured interviews of 30–60 minutes in duration between August 2022 and February 2023. These occurred via Zoom, Microsoft Teams, or telephone, based on participants’ preferences. While most interviews were conducted one-on-one, one interview had three participants, and two interviews had two participants each. Zoom and Teams audio recordings were professionally transcribed to help maintain participants’ original voices and allowed frequent revisiting of the data to maximise validity of the analysis. Detailed notes were taken during phone interviews as these were not recorded.

### Data analysis

Data were analysed using a rapid qualitative approach including the use of structured templates, purposeful data reduction and inductive-deductive analysis of matrix displays.^29,30^ How the six stages of rapid analysis were operationalised for data analysis in our study is described in Table 1.

**Table 1:** Six stages of rapid qualitative analysis and methods used to conduct each stage.

Rapid analysis stage	Methods applied
1. Creating a summary template	A summary template was developed in Microsoft Word aligned with the questions of the interview guide (Supplementary Material 2). A column for capturing quotes was also included.
2. Test driving the template with a transcript	The template was then tested by two researchers (BA and DB) who independently summarised the same two transcripts.
3. Amending the template and retesting	Summaries were compared and discussed, with the template found sufficient for the remaining analysis after clarification of some definitions.
4. Dividing the data and making summaries of the remaining data	The remaining transcripts were divided evenly between BA and DR for analysis. Each researcher independently summarised their allocated transcripts and spot-checked the other’s summaries.
5. Creating a matrix of summarised content	Summaries were grouped according to participants’ state/territory of practice and BA and DR worked together to reduce data into region-based matrices in Microsoft Excel. An inductive approach was used to identify unique follow-up pathways and services in each region (matrix columns). Data about a common set of pre-defined characteristics for each pathway/service were then deductively extracted (matrix rows), resulting in one matrix of follow-up pathways for each State/Territory (example in Supplementary Material 3).
6. Synthesizing data	We compared all region-based matrices to synthesize, categorize and visually represent common follow-up pathways across Australia.

To add depth to our findings and increase rigor, we performed methodological triangulation using additional data from online sources.^31^ We performed Google searches for each category of service/clinic identified within each geographical region to supplement the understanding of common pathways gained from participant interviews with publicly available organizational data. Data obtained from these searches were used to establish if services only mentioned in one or two jurisdictions were also available across other states and territories, as well as to fill gaps in service characteristics not provided in interviews. Finally, we used synthesised member checking^32^ to enhance data credibility and validity by presenting draft findings to a selection of national participants (*n* = 9) from each state and territory for feedback and further refinement. These participants were the lead study partner investigators in each state/territory (or nominated by these investigators) and had good knowledge of care in their jurisdiction.

## Results

Of the 123 invited stakeholders, 52 participants took part in the study (Table 2). Non-respondents were similar to respondents across key characteristics such as clinical discipline, location, and experience level. Forty-seven interviews were conducted: 43 via Zoom or Microsoft Teams, and four via telephone. Additionally, one participant provided brief written responses to the interview questions. Most participants were women and worked in metropolitan hospital-based settings, although regional, rural and remote areas were proportionally well represented. At least one participant was recruited from every Australian State as well as the Northern Territory (Supplementary Material 4: map of participant locations).

**Table 2:** Characteristics of interviewed participants (*n* = 52).

Demographic characteristic	Frequency (%)
**Gender**	
Women	39 (75)
Men	13 (25)
**Clinical Discipline or Role**	
Neonatologist	8 (16)
General Paediatrician	7 (13)
Clinical Nurse Consultant	6 (12)
Developmental Paediatrician	5 (10)
Occupational Therapist	5 (10)
Physiotherapist	4 (8)
Speech Pathologist	4 (8)
Paediatric Cardiologist	4 (8)
Psychologist	3 (6)
Social Worker	2 (4)
General Practitioner	1 (2)
Neuropsychologist	1 (2)
Other (hospital executive, disability manager)	2 (4)
**Organisational Role**	
Healthcare provider	26 (50)
Senior healthcare provider (programme, team, or clinical lead)	19 (37)
Executive or medical director	7 (13)
**Service Setting**	
Hospital	41 (81)
Community	11 (19)
**Service Location**^**a**^	
Metropolitan area	32 (62)
Regional centre	15 (29)
Rural area	2 (4)
Remote communities	1 (2)
State-wide	2 (4)
**State or Territory**	
Queensland	15 (29)
New South Wales	10 (19)
Northern Territory	9 (17)
Victoria	6 (12)
Western Australia	6 (12)
Tasmania	4 (8)
South Australia	2 (4)
**Total**	52

^a^Classified using the Modified Monash Model.

**Fig. 1:**
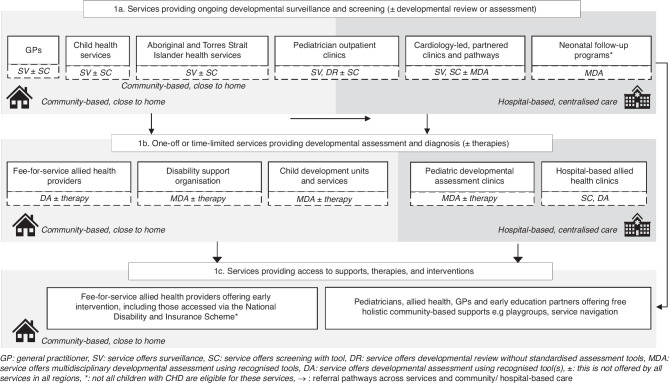
Graphical representation of the types of services and pathways providing neurodevelopmental follow-up care for children with congenital heart disease in Australia.

Our discussions with health service stakeholders across Australia indicated that children with CHD mostly receive neurodevelopmental follow-up by accessing a combination of existing services across the primary to quaternary healthcare spectrum rather than attending a dedicated cardiac follow-up programme (Fig. 1, Table 3). Stakeholders perceived embedding follow-up into existing health services in this manner to be an appropriate way to overcome resourcing and geographical challenges. The services and providers identified in our study can be categorised into three groups supporting follow-up: those which provide ongoing surveillance and screening across childhood (sometimes with assessment); services which provide targeted developmental assessment and identification (but do not provide ongoing follow-up); and services which provide early intervention and other supports. The availability of these services, and how families access them, varies across the country.

**Table 3:** Quotes from stakeholders describing a lack of structured cardiac neurodevelopmental follow-up in Australia.

Quote	Participant	Location
*“To be honest, there’s not a lot. There’s not a set pathway that’s for sure. I think the hospital probably uses a combination of referrals to a general paediatrician and a kind of a neurodevelopmental clinic in cardiology.”*	Clinical Psychologist	Victoria
*“We don’t have a dedicated or separate cardiac model [for developmental follow-up]. So, we access general services here for those purposes.”*	Paediatric Cardiologist	South Australia
*“There’s actually no real structured neurodevelopmental follow-up, other than if that’s recommended by us for them to go see a paediatrician.”*	Paediatric Cardiologist	Western Australia
*“Not at this stage, we don’t have a specific pathway, or any specific clinic set up for them [children with CHD]. So, there’s no pathway, really structured pathway. I think we probably couldn’t say we have a model of care. I don’t think we do.”*	Speech Pathologist	Northern Territory

Neurodevelopmental follow-up care for children with CHD in Australia therefore occurs largely though organisationally dispersed pathways, as opposed to programmes delivered by a single healthcare organisation. Structured follow-up programmes that do exist are not exclusively for children with CHD, but rather encompass a broader surgical or high-risk cohort. Participants also reported that each child follows a unique pathway based on their geographical location, medical and developmental needs, care preferences, and financial resources. Children do not always move linearly through these pathways, with referrals made between services and across levels of follow-up throughout their journey. However, the lack of formal structures to underpin and coordinate these care pathways for both services and families was a key concern for many of those interviewed. Participants in all states expressed that the lack of systematic follow-up could result in children without obvious developmental concerns at the outset failing to be identified for timely assessment and intervention.

“I feel that CHD—it’s disorganised—it’s not organised, not standardised.” [P46, Paediatrician, New South Wales]

“I think these kids come—they’re like—we deal with it case by case” [P29, Physiotherapist, Northern Territory]

### Services providing ongoing follow-up

Participants identified a range of services, in community and in hospitals, which were able to provide ongoing surveillance and developmental screening for children with CHD (Fig. 1a).

#### Community child health services and general practitioners

Every state and territory in Australia has a primary care pathway formally embedded in the community which is used for regular ongoing developmental surveillance of all children. This includes free, accessible child and family services led primarily by nurses for those 0-5 years old, as well as locally based general practitioners (GPs) who can provide additional surveillance beyond early childhood. First Nations children can also access culturally appropriate and community-controlled versions of these services. Follow-up record keeping is embedded in a child’s personal health record, a physical book remaining with the family to provide centralised documentation of their interactions across a variety of services in the first years of life. The personal health record has a focus on general developmental surveillance and health monitoring at standardised touchpoints. Formal developmental assessment is generally not undertaken but referrals can be made for assessment elsewhere if required. In some regions, participants reported the inclusion of developmental screening with standardised instruments such as the Parents’ Evaluation of Development Status (Queensland and Victoria), Ages and Stages Questionnaire (Northern Territory and Queensland) or “Learn the Signs. Act Early” milestones check (New South Wales). While the timing of follow-up varies across jurisdictions, regular touchpoints occur from two weeks old to pre-school age.

While community-based child health services and GPs do not exclusively follow-up children with CHD, many of those interviewed felt they were well placed to support foundational developmental surveillance and screening of these children, particularly those without any identified issues at the outset.

“Children that are low risk…are encouraged to continue screening with maternal and child health nurses and to raise concerns with them or GP should they arise.” [P45, Physiotherapist, Victoria]

“There’s a lot of time and a lot of support within that relationship with the maternal child health nurse.” [P26, Paediatrician, Northern Territory]

Participants also raised several perceived challenges with this model. Namely, that families are often responsible for remembering and organising a child’s follow-up, and that there may be limited collaboration between the surgical centre and community health services. Additionally, follow-up processes were reported to vary widely, as too the knowledge of community providers about the impact of CHD on development.

“When referring children out to community services, we have no control over how long or when they may be seen, it is up to each individual service. Some may only be seen once for a screen, some will be followed through closer over time. We also don’t have any control over what assessments may be used.” [P45, Physiotherapist, Victoria]

Several of those interviewed who worked in regions with large populations of First Nations’ children also perceived inequities between mainstream child health services and those which catered exclusively for this cohort. Finally, few study participants identified general practitioners as part of the pathway for regular developmental follow-up in Australia, however the presence of local champions was noted in interviews from participants in Queensland.

“Any child I see for any routine immunisation, I’m assessing their growth and development as just routinely a part of that.” [P4, GP, Queensland]

#### General paediatrics clinics

Regular follow-up and review in general paediatrician outpatient clinics was reported by participants in all jurisdictions as being a common pathway for delivering neurodevelopmental follow-up of children aged 0-18 years with CHD in Australia. This is because in many states children with CHD are linked with a paediatrician on hospital discharge and monitoring neurodevelopmental delay is part of standard paediatrician care. Additionally, families are often able to form long-term relationships with paediatricians, placing these health providers in a key position to provide oversight for ongoing developmental follow-up.

“I try and make sure I keep following these kids up over time. And so, I usually try and check in with them. I have a sort of system of following them up … even if they seem well, I try and catch up with them at least annually until they get to school… So, it’s a long-term relationship that you build with the family.” [P22, Paediatrician, Northern Territory]

While some of these clinics are delivered centrally at surgical centres, participants reported a large number to be delivered at smaller hospitals and sites in the community, including regular outreach to rural and remote areas. For this reason, paediatrician clinics were some of the most commonly reported follow-up pathways for regional centres of Queensland, Northern Territory, Western Australia, and Tasmania.

Despite being an important part of the pathway, the role of paediatric clinics in cardiac neurodevelopmental follow-up is not formalised, and processes lack standardisation across clinics and jurisdictions. For example, participants reported that the frequency of follow-up and timing of discharge is often based on the clinician’s discretion. Developmental reviews were reported to be embedded in general paediatric follow-up appointments with clinical expertise more often used for assessment compared to developmental screening measures.

“There’s over 20 paediatricians [in the region] and mostly, we do the same kind of work and follow the same kind of process, but potentially, 20 different children could have 20 different assessments or follow-ups that are just I suppose, beholden on that clinician’s individual experience and thought processes.” [P31, Paediatrician, Northern Territory]

Some paediatricians were also known among their colleagues as having a special interest in children with CHD and consequently, their clinics were seen as key drivers of referral and follow-up in that region.

#### Cardiology led or embedded services

At hospitals in Sydney, Brisbane, and Melbourne, we identified neurodevelopmental and psychological services led by cardiology units, often in partnership with other healthcare providers. These were each very different, comprising a cardiac psychology programme, developmental screening embedded within cardiology review, and a community-based follow-up pathway including developmental screening with primary care services. Table 4 provides descriptions of how these services are operationalised in each setting. The cardiac psychology programme in Sydney was reported to work in partnership with the neonatal surgical follow-up programme based in the same health service (Grace Centre for Newborn Care, see Supplementary Material 5) to expand the eligible cohort of that programme, as well as provide additional types of services and therapies. While cardiology services in other regions likely play a role in developmental surveillance of children with CHD, participants did not report any others using similar formalised processes or embedded pathways. Rather, follow-up was more ad hoc and reliant on individual cardiology providers.

**Table 4:** Characteristics of neurodevelopmental follow-up care provided by cardiology services.

State	Victoria	New South Wales	Queensland
**Name of pathway/service**	Cardiology outpatient clinic	Cardiac psychology service	Congenital Heart Disease Long-term Improvements in Functional Health (CHD LIFE) pathway
**Where is it based?**	Royal Children’s Hospital, Melbourne	Embedded in the Sydney Children’s Hospitals Network (SCHN) Heart Centre for Children, Sydney	Queensland Children’s Hospital, Brisbane, with care delivered across the state
**Which clinical service leads it?**	Cardiology	Cardiac Psychology	Cardiology and allied health
**What is it?**	Developmental screening embedded into Cardiology review: “*When they come to clinic there is a trigger in our electronic record that generates the survey and the parents fill it out, and then we score it and then either act on those scores or refer them to our team for follow up after that.” [P36, Paediatric Cardiologist]*	Cardiac psychology service works in partnership with the Grace Centre for Newborn Care (neonatal unit in same hospital network – see Supplementary Material 5*) to provide neurodevelopmental evaluation and care, and comprehensive psychological services across the medical trajectory (including screening, assessment, intervention, and treatment) *“We provide a range of different kinds of services. So psychological screening, assessment, a range of different therapies - whether that be for the baby, child, adolescent or adult heart disease, their parents or caregivers, their siblings, their grandparents, the family as a whole.” [P14, Psychologist]*	State-wide follow-up pathway in partnership with health services and primary care: *“It’s a primary surveillance model with referral on if there are difficulties detected to a more specialised service, like a multidisciplinary integrated child development service” [P23, Paediatrician]*. Focus on providing families with education and empowerment to access follow-up support independently.
**Which babies/children are eligible?**	All children returning for Cardiology follow-up.	All children with paediatric or congenital heart disease and their families can access psychological support and mental healthcare. Children with CHD who have undergone cardiac surgery before age 12-months can access neurodevelopmental evaluation via the cardiac psychology service. The specific cohort of children with CHD who are admitted to the NICU after neonatal surgery are systematically enroled and followed-up at regular intervals in the Grace Centre’s Development Clinic (Supplementary Material 5). State-wide remit.	All children with CHD who have surgery at the hospital in the first year of life. State-wide remit.
**How does follow-up care commence?**	Systematically embedded in standard Cardiology follow-up for all children with CHD.	Psychology service systematically contacts all eligible families to offer evaluation and services. Accepts referrals from a wide range of sources including medical specialists, parents, nurses, and allied health professionals. Do not need a previous admission at the hospital to be eligible.	Systematic referral of children onto pathway. Hospital-based allied health team refer on to community services for regular surveillance and screening, and assessment and intervention as indicated. Medical team refer to paediatricians. Consenting families entered into pathway database.
**How is service delivery structured?**	Centralised at hospital. In-person.	Centralised at hospital. In-person services. Telehealth may also be offered, depending on the nature of the referral, clinical presentation, and therapies needed.	Decentralised, care close to home. In-person. Some services use telehealth.
**Who delivers follow-up?**	Cardiologist and physiotherapist	Psychologists in collaboration with a multidisciplinary team.	Various, with a focus on primary health care providers (child health nurse, GPs, aboriginal health workers, allied health)
**What are the follow-up procedures and measures used?**	Developmental survey (Ages and Stages) triggered in electronic medical record at specific Cardiology reviews. Survey completed by parent and collated and reviewed by physiotherapist. Referrals to GP or for formal assessment in community as needed.	Psychological screening, assessment, and a range of therapies for infants to adults. The Grace Centre’s Development Clinic provides multidisciplinary developmental and medical assessment for the eligible cohort (see Supplementary Material 5).	Family education and awareness about follow-up before discharge. Regular developmental surveillance and screening in community/primary health care encouraged using Ages and Stages Questionnaire. Refer on for formal assessment or intervention as needed. Follow-up can look different across the state but principles are the same: *“There’s a referral pathway which takes them back to those local hospital and health services to follow up. But then how’s that executed on the ground is variable” [P17, Executive Director]*.
**When is follow-up is provided?**	Survey flagged at 12 months, 2 years, and pre-school age	Cardiac psychology services can be provided from pre-natal diagnosis to age 18 years. No age restrictions for caregivers. The Grace Development Clinic sees children regularly to 3 years of age.	Surveillance and screening at 6 months, 12 months, 18 months, 2.5-3.5 years, 4-5 years, 11-12 years, and 15 years as a minimum.
**Additional information**		Both clinics are funded through combination of health service, research grants, and philanthropy contributions	Involves capacity building and education of community providers

*relevant information about how Grace Centre for Newborn Care works together with the cardiac psychology service is presented here, while more detailed information is presented in the Supplementary Material with the other neonatal programmes.

*CHD* congenital heart disease, *GPs* general practitioner, *NICU* Neonatal Intensive Care Unit, *PEDS* Parents’ Evaluation of Developmental Status.

“It is reliant on cardiologists and cardiac nurses identifying the need for that additional [developmental] input rather than having a structured programme that just kicks in automatically and supports those kids and families.” [P39, Paediatric Cardiologist, South Australia]

#### Neonatal follow-up programmes

Participants identified four major hospital centres which have eligibility criteria within established neonatal follow-up programmes to allow additional inclusion of some high-risk children with CHD. For example, programmes based at hospitals in Western Australia, Tasmania and New South Wales enrol all children with CHD who have surgery or intervention in the first three months of life. In Darwin, enrolment of children with CHD in neonatal follow-up occurs on ad hoc basis, although plans are in place to formalise this process.

“So, we’ve come up with a criteria that if they’ve [children with CHD] undergone major cardiac surgeries in the first three months of life…we would be happy to link them in our developmental follow-up programme.” [P1, Neonatologist, WA]

Children with CHD undergoing follow-up in these programmes were reported to receive regular medical and developmental assessment from a multidisciplinary team, including the use of formal assessment measures (e.g., Bayley Scales of Infant and Toddler Development). However, most of these clinics did not routinely offer follow-up beyond four years of age. Given that neonatal follow-up programmes were reported to have a state-wide remit and usually incorporated systematic referral and enrolment of children from within the hospital, they are well placed to capture the cohort of children with CHD who would be eligible for these services. However, they also require families to travel to the hospital to receive care, although both postal questionnaires and telehealth were reported to be used occasionally at timepoints where formal assessment may not be needed. Detailed characteristics of these programmes are provided in Supplementary Material 5.

### Services providing targeted or time-limited follow-up

Interviews highlighted an additional group of services within follow-up pathways that were responsible for formal evaluation and identification of concerns for children with CHD (Fig. 1b). The majority of these services were located in the community.

#### Child development and assessment units

Publicly funded child development services/units featured predominantly in our discussions with healthcare professionals when considering follow-up pathways for children with CHD in Australia. These types of services do not provide ongoing surveillance for children, as referrals are only accepted once developmental concerns have been identified. However, they were considered by participants to play an important role in the formal assessment, identification, and diagnosis of developmental delays and disorders in this cohort. Additionally, interaction with child development services is a necessary step for many children to access intervention and support. Aside from referral prioritisation in some regions, these child development units were not reported to deliver specialised care pathways or services for children with CHD.

“There isn’t a pathway between our cardiac surgery and cardiology services into our developmental service. So really, it’s fairly ad hoc and for a specific question …or behavioural concerns.” [P8, Developmental Paediatrician, Victoria]

Child development services are mostly delivered face-to-face in community settings, providing care closer to home for families. In Queensland, participants also described a recently established virtual model of child development whereby clinicians in regional areas can partner with an experienced centralised team via telehealth.

Services are usually staffed by a multidisciplinary team of health providers including paediatricians, nurses, and a range of allied health professionals. This enables them to provide a comprehensive multidisciplinary developmental assessment considering functional outcomes and formal diagnoses. Very few of these services are able to provide intervention or therapy, but all support planning and referral for families to access these as required.

“The bulk of our work around child development is we understand where the child’s development is at this point in time. We help the family understand what that looks like and help them to advocate [for intervention and support].” [P25, Speech Pathologist, Queensland]

#### Hospital-based paediatric developmental assessment clinics

Participants reported centralised hospital-based developmental assessment clinics to be mostly reserved for children with complex or chronic conditions due to capacity issues. These are generally staffed by developmental paediatricians with or without allied health input. Processes include triage, intake, family education, formal assessment, and referral to other services and intervention as needed. Once again, children are not systematically referred for developmental follow-up, only after identified concerns. Our research suggests this part of the follow-up pathway serves only a small proportion of children with CHD.

#### Hospital-based allied health clinics

A small proportion of children with CHD were reported to be referred to hospital-based allied health clinics where they may be receiving care or follow-up for specific concerns (e.g. feeding issues). During their time with these services, they often receive screening or assessment, although it is not often multidisciplinary. Reported examples include a speech pathology clinic in Perth which provides regular communication screening and a psychology clinic in Melbourne, where children with developmental concerns can receive a mental health and developmental assessment. Additionally, a physiotherapy clinic in Perth performs standardised gross motor assessment for children with CHD. However, children only stay with these services for defined period, limiting their ability to provide ongoing surveillance and screening.

#### Private community providers

In each state and territory, participants reported a range of community-based providers which offer formal developmental assessment and diagnosis for children outside the public health system. These are generally fee-for-service clinics led by developmental paediatricians, psychologists, or psychiatrists. While families do have to pay out of pocket costs for these services, wait times are generally shorter. Many also provide therapy or intervention for children with identified concerns. While participants in our interviews acknowledged the importance of being able to access private providers as part of cardiac developmental follow-up pathways, it is unclear what proportion of follow-up is currently undertaken in the private system.

### Services providing intervention, including therapy or other support

Participants expressed that all services and providers across the follow-up pathway have an explicit focus on supporting families to access the most appropriate service to support their needs. Children with CHD are referred for early intervention and/or other ongoing support depending on their developmental status, diagnosis, needs and preferences (Fig. 1c). Within Australia, the type of intervention or support service children can access is also dependant on age and funding eligibility.

Participants reported intervention and therapies for children to be provided by a range of allied health clinicians such as speech therapists, occupational therapists, psychologists, and social workers. Many children with CHD and developmental delay access intervention services via this part of the pathway. These providers are almost always based in the community and charge a fee for service unless the child is accessing services via the National Disability Insurance Scheme (NDIS). The NDIS is a government funded service to support the costs associated with disability. While CHD alone is not a qualifying criterion, the NDIS does have a criterion for functional developmental delay which can be met by children up to six years of age without evidence of permanent lifelong disability. Once deemed eligible for this scheme, children can access a variety of allied health supports at no cost. Consequently, for a proportion of children with CHD, particularly those with early identified delays, qualifying for the NDIS is an important part of the follow-up pathway in Australia.

Aside from early intervention and therapies, families can also access holistic supports in the community under a nationwide network of paediatrician, allied health, GP, and early education partners. These include free developmental playgroups, child health and development workshops, holistic family support, service navigation, and day care assistance for children up to 8 years old. Several participants highlighted these supports as an important supplementary pathway to support children with CHD who have milder developmental concerns or do not qualify for the NDIS, as families can self-refer, and a formal diagnosis is not required.

“Yeah, I think it’s fantastic. And I think a lot of those foundational pieces, I think are always overlooked and it’s easier to step towards more of the intervention approach.” [P28, Paediatrician, Northern Territory]

## Discussion

Our national interview study of healthcare professionals revealed that neurodevelopmental follow-up care for children with CHD in Australia is primarily delivered as pathways through existing services rather than standalone centre-based cardiac follow-up programmes. Predominantly, follow-up care is shared across services best equipped to deliver each component, with community-based primary care services, paediatric clinics, child development services, and allied health providers the most commonly reported touchpoints for follow-up of children across the country. Pathways through these services can provide families with all necessary components of cardiac developmental follow-up care. Notably however, as management of health services falls under different jurisdictional governance, the contribution of each type of service to follow-up pathways varies across the country. Additionally, there was a consistent view among participants that systematic coordination of care pathways and greater structure both within and across jurisdictions is likely required to optimise care.

Australia’s pathways of cardiac neurodevelopmental follow-up care differ from the predominance of cardiology-led, hospital-based models of care reported in international literature. Surveys have estimated that almost 70% of US-based cardiology services have access to CHD specific, hospital-based neurodevelopmental follow-up programmes.^8,33,34^ Likewise, similar programmes are available at 40-50% of surgical centres in Canada and Europe.^10,11^ The contrast observed in our study was not an unexpected finding, as geographical, financial, health system, and capacity constraints have previously been highlighted as key challenges to implementing centralised follow-up care programmes in the Australian context.^15,35^ Research from Canada and the United Kingdom has also highlighted concerns about the resourcing implications and viability of adopting centralised models in their healthcare contexts.^10,14^ Consequently, our study identifies alternate options for care delivery and how follow-up processes can be adapted to meet local needs and contexts.

Despite the limited number of cardiac specific programmes and services, all Australian jurisdictions recognise the importance of neurodevelopmental care, and have tried to incorporate other approaches to follow-up. For example, a proportion of children with CHD access formalised follow-up via neonatal programmes. This embedded and integrated approach has also been adopted in numerous services in the US,^33^ Canada^10^ and Switzerland.^36^ Expanding access to these types of established programmes in Australia provides one option for more formalised follow-up of infants and younger children with CHD whose families have a preference and capacity to visit tertiary and quaternary centres for care.

Additionally, interviews only identified one Australian centre which has systematically incorporated developmental surveillance into regular cardiology review, yet 75% of cardiologists surveyed in South Africa routinely perform developmental surveillance and screen with standardised measures.^13^ This practice is also common for US and Canadian based cardiology services without access to a formal follow-up programmes.^8,10^ Moreover, an increase in developmental referral numbers with standardised and systematic surveillance during cardiology appointments has been observed.^37^ Given that children with CHD in Australia regularly visit cardiologists at both tertiary and more locally-based hospitals, increasing developmental surveillance within cardiology services could be considered a key strategy for improving neurodevelopmental follow-up. If processes and measures are agreed at a national level, it would represent a valuable opportunity for better care standardisation across the country. Further investigation of the feasibility and acceptability of this approach is needed, including its application to outreach, and privately operated cardiology clinics.

In addition to these specialist-based services, our research has also highlighted the key role that paediatric clinics and the primary care system play in the neurodevelopmental follow-up of children with CHD in Australia. These providers are well placed to care for this cohort due to their accessibility, regular interactions with families, knowledge of local services, and high frequency of developmental screening in their practice.^13,38^ The belief that primary care physicians and paediatricians are well-suited to undertake developmental surveillance, screening, and referral is frequently reported in international literature.^10,34,39,40^ However, the importance of these services in providing foundational support to cardiac neurodevelopmental follow-up care models has received insufficient recognition. Moreover, research suggests that primary care providers are not adequately supported with guidance or communication from cardiologists, particularly about specific developmental concerns of children with CHD under their care.^8,38,41^ Additionally, formal neurodevelopmental assessment measures are less frequently used within this setting^13,42^ and referrals for additional evaluation are much more reactive to concerns, rather than proactive as recommended in AHA guidelines.^13,38^ If primary care services are to fulfil their potential in supporting the developmental follow-up of children with CHD, increased developmental training opportunities and collaboration with cardiology services are likely required.

### Alignment with best practice and future directions

The recently published Australian National Standards of Care for Childhood-onset Heart Disease provide a blueprint for Australian paediatric cardiac services to inform care delivery that maximises the health and wellbeing of individuals living with CHD and their families in the unique Australian environment.^7^ Informed by existing recommendations,^6,43,44^ the standards support the need for a whole-of-life approach to neurodevelopmental and neurocognitive care. This includes the need for universal access to standardised developmental screening, and ongoing formal developmental and medical evaluation for all children at high-risk of developmental delays/disorders, with access to evidence-based interventions, services, and supports. Coordinated models of care that include primary care, specialist, and family partnerships are essential for optimal care delivery.

Participants in all jurisdictions recognised the importance of providing long-term developmental follow-up for children with CHD, using the varied but sometimes limited resources available in each centre. However, this study highlights that recommendations in the Standards are currently not routinely delivered, with barriers to this in the Australian context previously reported.^12,41^ Furthermore, the main concerns raised by participants related to improving the consistency and continuity of care while also ensuring all eligible children are captured. Future approaches to improve neurodevelopmental follow-up care in Australia will benefit from integrating, leveraging, and growing existing services, although adopting new models may also be needed. Additionally, clarification of roles and responsibilities for services and providers, along with more systematic and structured processes for moving through care pathways is required. These will be important considerations which inform the development of clinical guidelines, workforce requirements, and infrastructure planning.

### Strengths and limitations

This was a national study with a large and diverse sample of health service participants, facilitating collection of a comprehensive range of perspectives about cardiac neurodevelopmental care services from across the country. However, despite repeated recruitment attempts, only a small number of the participants were GPs and cardiologists. Therefore, the views of these professionals may not be as prominent as others in the findings. While the response rate across all those invited was only 42%, this is similar to health professional response rates in other qualitative studies conducted online.^45^ As invitations were made by study partners in each jurisdiction it was not clear if those invited refused participation, or simply could not be contacted, making it harder to examine this potential limitation further. As the aim with recruitment was to cast a broad invitation across a range of relevant stakeholders rather than target groups familiar with the investigator team, our response rate is somewhat expected, and characteristics of non-responders did not different from study participants.

Importantly, the qualitative design did not just capture what services were offered but also allowed an in-depth exploration of other considerations such as their suitability or key challenges. However, it could be argued that using a qualitative design to perform service mapping may have limited the list of follow-up services in our study to those within the knowledge of those interviewed. To address this concern, we supplemented interview data with web-based searches in the late analysis stages to confirm the presence or absence of follow-up services in each region. Nevertheless, other less frequently used services may exist on national care pathways. Finally, the attainment of data saturation, and use of transcripts, verbatim quotes, clinician team members, and member checking strengthened the rigour of our findings.

## Supplementary information

Supplementary Information

## Data Availability

Data are not publicly available due to information that could compromise the privacy of research participants if published. However, excerpts of the transcripts relevant to the study that support the findings are available on request from the corresponding author.
