# Supplementary material for: Neurodevelopmental follow-up care pathways and processes for children with congenital heart disease in Australia
Source: Pediatr Res. 2024 Nov 23;98(3):966–75. doi: 10.1038/s41390-024-03722-2 (PMC12507673; doi:10.1038/s41390-024-03722-2)
Supplement: Supplementary file 1 — Supplementary Information [file 41390_2024_3722_MOESM1_ESM.pdf]

Supplementary Material 1: CHD LIFE+ Semi-Structured Interviews with Providers and Stakeholders

Aims:

- Understand the current models of care in place nationally that are supporting the long-term neurodevelopmental needs of Australian children with congenital heart disease

Structure:
The interviews will be semi-structured, with open-ended questions which are adaptable to reflect the responses being provided. The conversation should explore issues as they are raised, with guidance and prompting as required. Interviewers/facilitators will ensure discussion progresses in a timely, yet informative manner. Interviews will last no more than 60 minutes, focus groups will last a maximum of 90 minutes.

Content:
Review aims of session, participant information sheet, particularly confidentiality, consent, privacy and right not to participate. Begin recording and ask for verbal consent (virtual/phone) or check and collect signed consent forms (in person). The content of the interviews will be based on the models of care and characteristics found during evidence mapping.

Guide:

Great, so I just wanted to start by learning a bit about you and your experience with congenital heart disease and follow-up care.

- Could tell me a bit about your current role and how it's related to supporting children with congenital heart disease? *(Probes: How long?...How get involved?)*

So, I'm keen to hear about what type of pathway or model of care you are currently using to support the neurodevelopmental follow-up of children with congenital heart disease.

- Can you talk me through what that looks like? *(Probes: eligibility, service location, components, frequency of review, tools/assessments, costs, tailoring, complexity, providers, evidence-base, adaptability)*

- Can you talk me through the process a child/family might go through to access and engage with your service? *(prompts: so what happens next? what about any care transitions? Referrals out to other services and collaboration?)*

Can you tell me about any other types of care pathways/models you are aware of in Australia? (*probes: would you prefer to use those here? Why/why not?)*

Have you seen or read about any other pathways/models of care internationally that you think would work well for your setting?

a. If yes, tell me about them and why they would be beneficial
b. If no, well some examples are hybrid phone and in-person models, decentralised clinics, psychologist led etc. What do you think about the suitability of any of those?

Do you have any questions for me?

Thank you for participating in the interview today. Please feel free to get in contact with the research team if you have any questions. We're also planning to hold a national workshop in a few months' time. Would you be interested in hearing about that?

[end of interview]

Supplementary Material 2. Template for rapid qualitative analysis of each transcript

<table>
<tr><th colspan="2">Summary prepared by:</th><td colspan="2"></td></tr>
<tr><th colspan="2">Interview number:</th><td colspan="2"></td></tr>
<tr><th colspan="2">Number of participants:</th><td colspan="2"></td></tr>
<tr><th colspan="2">Participant state:</th><td colspan="2"></td></tr>
<tr><th colspan="2">Participant role(s):</th><td colspan="2"></td></tr>
<tr><th colspan="2">Other relevant participant information:</th><td colspan="2"></td></tr>
<tr><th colspan="2">PART 1</th><td colspan="2">Understand the current models of care in place nationally that are supporting the long-term neurodevelopmental needs of Australian children with congenital heart disease</td></tr>
<tr><th></th><th>Question</th><th>Summary of key themes/response</th><th>Quotes/line numbers</th></tr>
<tr><th rowspan="2">Australian ND pathways</th><td>What type of pathway or model of care you are currently using to support the neurodevelopmental follow-up of children with congenital heart disease?

Can you talk me through the process a child/family might go through to access and engage with your service?</td><td>Description of current care pathways in own jurisdiction/state for supporting long-term neurodevelopmental needs and their key characteristics.

Code here: eligibility, service location, components, frequency of review, tools/assessments, costs, tailoring, complexity, providers, evidence-base, adaptability, transitions referrals, collaboration, how access etc</td><td>Example quotes or references to further sections for more detail</td></tr>
<tr><td>Can you tell me about any other types of care pathways/models you are aware of in Australia?</td><td>Description of care pathways in other states that may be useful/worth considering – include why/why not work in context.

Code here: eligibility, service location, components, frequency of review, tools/assessments, costs, tailoring, complexity, providers, evidence-base, adaptability, transitions referrals, collaboration, how access etc</td><td>Example quotes or references to further sections for more detail</td></tr>
<tr><th>Other</th><td>N/A</td><td>Summarise any other key discussion points here</td><td>Example quotes or references to further sections for more detail</td></tr>
</table>

Supplementary Material 3: Example of a state-based matrix of care pathways

Table SM1. Regional data analysis matrix for pathways of care observed in Western Australia. Columns are unique services and rows are characteristics of each service.

State	**Northern Territory (n=8)**				
Pathways reported	**Pathway 1**	**Pathway 2**	**Pathway 3**	**Pathway 4**	**Pathway 5**
Type of pathway	**Developmental follow-up clinic (TEDI)**	**General paediatrics clinic**	**Community-based surveillance**	**Community-based assessment**	**Cultural and community-based surveillence**
**Where?**	Centralised: Royal Darwin Hospital	Centralised: Royal Darwin Hospital	Community: public health services	Community: public health services	Community: aboriginal health services
**Clinical service**	Neonatal unit	Paediatrics clinic	Maternal and child health	Child development service	Aboriginal Health Service
**Eligibility: for who?**	Babies born pre-term, low birth-weight or had neurological insult. CHD kids only qualify if meet those criteria - rarely.	All children but is a specialised service.	All children	All children ages 0-18 in urban Darwin	All First Nations children
**Enrolment**	Systematically referred as hospital inpatient, or as handover from interstate returns. Paediatricians.	Via GPs or community child development AH team, Cardiologists, ED. Sometimes schools or child health nurse.	Walk-in/access	Via neonatal follow-up program or other sources	GP referrals
**How?**	Face to face	Face to face	Face to face	Face to face	Face to face
**Who delivers it?**	Paediatricians and neonatologists. MD follow-up with physiotherapist, ST, dietician.	Paediatricians - care may vary depending on which paediatrician running clinic	Nurse	Allied health team	
**What procedures?**	Comprehensive MDT assessment of gross motor, fine motor, communication and feeding. With physiotherapist, OT, ST, dietician and neonatologist. Little bit of parental education.	Not standardised just individual practice in clinics. No tools just judgement. Growth and development part of normal clinic. Mostly ongoing ND care rather than monitoring and screening. Refer out to community for ongoing care.	Maternal and child health checks, developmental assessment and ASQ during vaccinations. Not specific CHD knowledge.	Therapeutic interventions or assessments where there are concerns.	Intake, multidomain check-up, refer for assessment elsewhere
**When and how muc**	3 months, 6-8 months, 12 months, 2 years, 4 years	Each paediatrician would have their own schedule			
**Other**	Currently not funded. Working to embed CHD kids at just 6-8 month review.				

ASQ, Ages and Stages Questionnaire; CHD, congenital heart disease; ED emergency department; GP, general practitioner; MD, multidisciplinary; ND, neurodevelopmental; OT occupational therapist; ST, speech therapist.

Supplementary Material 4: Map highlighting the locations of participants interviewed for the study

Note: Multiple participants were located in Brisbane, Gold Coast, Townsville, Sydney, Melbourne, Perth, Darwin and Launceston.

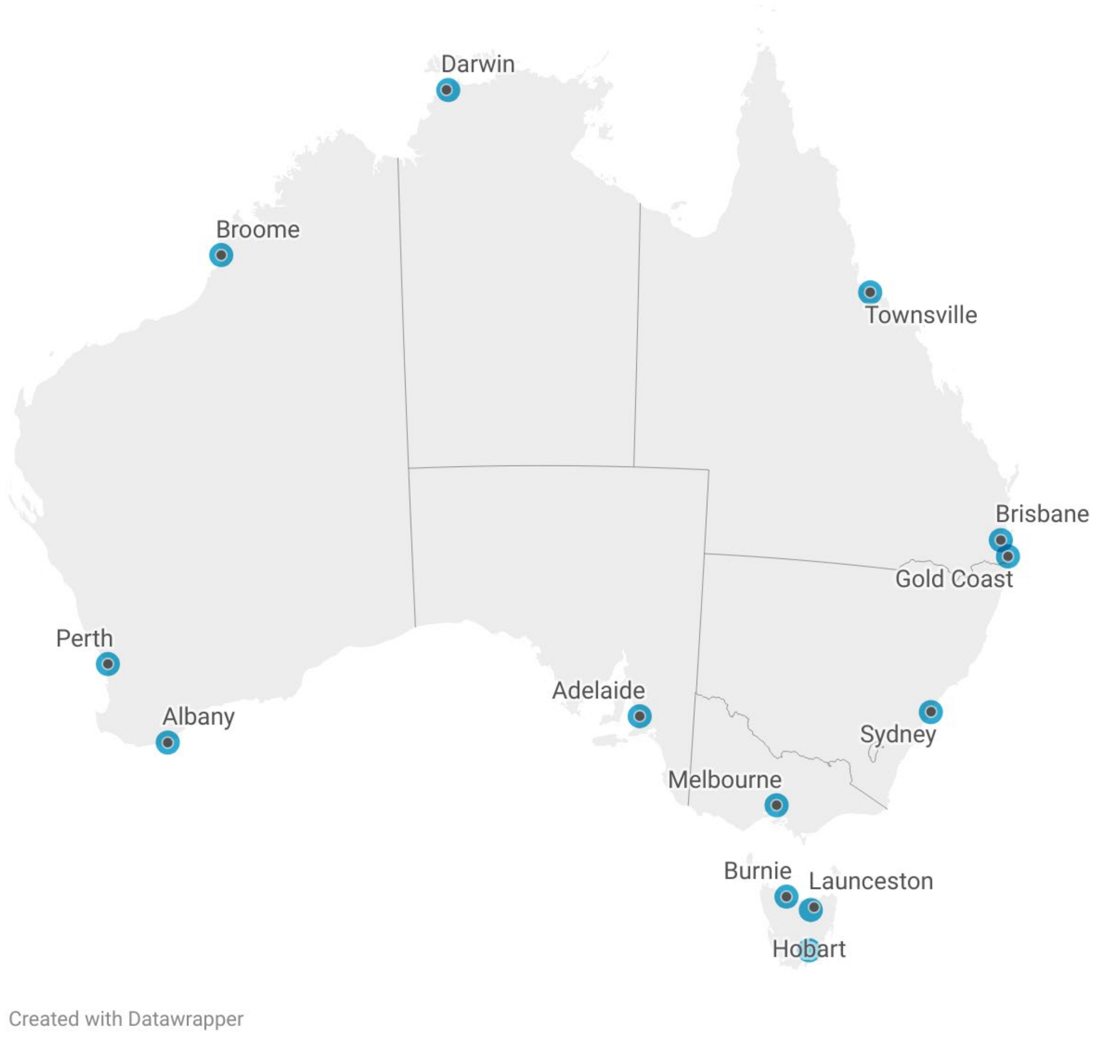

Figure A1. Cities in which interviewed participants were located (n=52)

Supplementary Material 5: Examples of neonatal follow-up programs supporting children with CHD in Australia

Table A2. Characteristics of neonatal follow-up programs supporting children with CHD

State	Western Australia	New South Wales	Northern Territory	Tasmania
**Neonatal follow-up program name and location**	Neonatal follow-up program, Perth Children's Hospital and King Edward Memorial Hospital, Perth	Grace Development Clinic, The Children's Hospital Westmead, Sydney	Top End Developmental Infant Clinic, Royal Darwin Hospital, Darwin	Early Development Clinic Royal Hobart Hospital, Hobart
**Which clinical service leads it?**	Neonatal unit	Grace Centre for Intensive Newborn Care (Neonatal unit)	Allied health and paediatrics	Paediatrics
**Which babies/children are eligible?**	All high-risk children (born pre-term, low birth weight, surgery at less than 28 days old). The program also accepts children with CHD who have surgery up to 3 months of age. State-wide remit.	Infants who have undergone major surgery in first 3 months of life, or with neurological risk factors. The program also accepts children with CHD who have been admitted to the NICU after undergoing neonatal surgery/intervention. Children with CHD make up 40% of cohort. State-wide remit.	All high-risk children (pre-term, low birth weight). The clinic has also been accepting some children with CHD on an ad hoc basis at clinicians' discretion. Plans to formally add CHD to eligibility criteria (just for 6-8 month review). Covers Top End of Northern Territory.	All high-risk children (pre-term, low birth weight, hypoxic). The clinic also accepts children with CHD who have surgery up to 3 months of age (classed as medium risk in clinic).
**How are children enrolled?**	Systematic referral of all eligible children via central liaison (links the cardiac and developmental services). Via the hospital or local cardiologist.	Systematic enrolment of eligible infants following discharge from Grace NICU. Capture almost all children with CHD who have surgery in the neonatal period. Occasional referrals accepted from hospital-based occupational therapist, paediatrician, cardiologist or PICU.	Not systematically referred. For children with CHD, paediatricians can refer in children.	Embedded clinical pathway. Systematic referral and enrolment of all eligible children from neonatal unit, paediatrics team or referred in by clinicians in community or hospital.
**How is service delivery structured?**	Centralised at hospital. Mixture of in-person and postal questionnaire-based follow-up. Option to transfer follow-up to regional providers for those living far from centre.	Centralised at hospital. In-person. Some capacity for telehealth as needed for rural/remote families.	Centralised at hospital. In-person.	Centralised at hospital. In-person. One check-in phone call at 12-month follow-up.
**Who delivers follow-up?**	Neonatologist, psychologist	Physiotherapist, occupational therapist, neonatologist	Physiotherapist, occupational therapist, speech pathologist, neonatologist	Paediatrician, speech pathologist, physiotherapist, and occupational therapist
**What are the follow-up procedures and tools used?**	Formal developmental assessment including use of Griffiths (at 12 months) and Bayley (at 2 years). Ages and Stages Questionnaire sent out to parents at all timepoints. Refer out to other community services for further assessment, therapy, or intervention.	Multidisciplinary developmental and medical assessment. Formal assessment with Bayley, Hammersmith Infant Neurological Examination and General Movements Assessment. Refer to other services in hospital or community for therapy or intervention. Referral to NDIS or speciality service providers such as Cerebral Palsy Alliance.	Multidisciplinary developmental and medical assessment. Formal assessment with Bayley. Parental education.	Multidisciplinary developmental and medical assessment. Formal assessment with Hammersmith Infant Neurological Examination (3 months), General Movements Assessment (3 months), Modified Checklist for Autism in Toddlers Revised (18 months), Parent Report of Children's Abilities Revised (2

				years). Screen for red flags (e.g. delayed milestones) (9 months).
**When is follow-up is provided?**	At 4 months (postal), 8 months (postal), 12 months (in-person) and 2 years (in-person). Looking to extend to 5 years.	At 3-4 months, 12 months, 3 years. Refer to ongoing follow-up in community after age 3.	At 3 months, 6-8 months, 12 months, 2 years, 4 years	For CHD children (medium risk): 3-4 months, 9 months, 18 months, 2 years

CHD, congenital heart disease; NDIS, National Disability Insurance Scheme; NICU, neonatal intensive care unit; PICU, paediatric intensive care unit
